# Persistent Crimean-Congo hemorrhagic fever virus infection in the testes and within granulomas of non-human primates with latent tuberculosis

**DOI:** 10.1371/journal.ppat.1008050

**Published:** 2019-09-26

**Authors:** Darci R. Smith, Charles J. Shoemaker, Xiankun Zeng, Aura R. Garrison, Joseph W. Golden, Christopher W. Schellhase, William Pratt, Franco Rossi, Collin J. Fitzpatrick, Joshua Shamblin, Adrienne Kimmel, Justine Zelko, Olivier Flusin, Jeffrey W. Koehler, Jun Liu, Kayla M. Coffin, Keersten M. Ricks, Matt A. Voorhees, Randal J. Schoepp, Connie S. Schmaljohn

**Affiliations:** 1 Virology Division, U.S. Army Medical Research Institute of Infectious Diseases, Ft. Detrick, Maryland, United States of America; 2 Diagnostic Systems Division, U.S. Army Medical Research Institute of Infectious Diseases, Ft. Detrick, Maryland, United States of America; 3 Pathology Division, U.S. Army Medical Research Institute of Infectious Diseases, Ft. Detrick, Maryland, United States of America; 4 Headquarters Division, U.S. Army Medical Research Institute of Infectious Diseases, Ft. Detrick, Maryland, United States of America; Karolinska Institute, SWEDEN

## Abstract

Crimean-Congo hemorrhagic fever (CCHF) is the most medically important tick-borne viral disease of humans and tuberculosis is the leading cause of death worldwide by a bacterial pathogen. These two diseases overlap geographically, however, concurrent infection of CCHF virus (CCHFV) with mycobacterial infection has not been assessed nor has the ability of virus to persist and cause long-term sequela in a primate model. In this study, we compared the disease progression of two diverse strains of CCHFV in the recently described cynomolgus macaque model. All animals demonstrated signs of clinical illness, viremia, significant changes in clinical chemistry and hematology values, and serum cytokine profiles consistent with CCHF in humans. The European and Asian CCHFV strains caused very similar disease profiles in monkeys, which demonstrates that medical countermeasures can be evaluated in this animal model against multiple CCHFV strains. We identified evidence of CCHFV persistence in the testes of three male monkeys that survived infection. Furthermore, the histopathology unexpectedly revealed that six additional animals had evidence of a latent mycobacterial infection with granulomatous lesions. Interestingly, CCHFV persisted within the granulomas of two animals. This study is the first to demonstrate the persistence of CCHFV in the testes and within the granulomas of non-human primates with concurrent latent tuberculosis. Our results have important public health implications in overlapping endemic regions for these emerging pathogens.

## Author summary

CCHF is an emerging tick-borne viral disease that is endemic across much of Africa and Asia, and parts of Europe where its range and exposure risk to human populations is expanding. Tuberculosis threatens millions of lives world-wide and is the leading cause of death due to a bacterial pathogen. Concurrent mycobacterial infection with other infectious diseases has been described, but not for CCHFV despite the geographic overlap of these two pathogens. During our study we unexpectedly determined that some of the animals had latent tuberculosis and that CCHFV can persist within the granulomas. Furthermore, our study provides the first direct evidence that CCHFV can replicate and persist in the male genital tract, which has important implications for human sexual transmission. The ability of viral RNA to persist in immune-privileged sites or fluids has been described with increasing frequency for other emerging infectious diseases and can cause a burden on public health. This provides the impetus to utilize the model described here to better understand the mechanisms of CCHFV persistence and its effect on the development of long-term sequelae.

## Introduction

Crimean-Congo hemorrhagic fever virus (CCHFV) is a tick-borne member of the *Nairoviridae* family in the RNA virus order *Bunyavirales*. Its extensive range includes Africa, the Balkans, the Middle East, Russia and western Asia, and this range is thought to be continuously expanding as a result of both climate change and ecological disruption [1, 2]. CCHFV is maintained in nature through vertical and horizontal transmission cycles by several genera of ixodid ticks, which transmit the virus to a variety of wild and domestic animals that may be viremic without signs of illness. Human infections occur primarily by tick bites or exposure to blood or bodily fluids from infected animals or CCHF patients. *Hyalomma* ticks are the principal source of human viral infection and a subsequent disease state that can range in severity from a mild, non-specific febrile illness to a lethal infection characterized by hemorrhagic manifestations. Patient data and animal studies show that after an initial local replication, the virus spreads systemically and targets the liver and endothelium where it causes a massive dysregulation of the immune response sometimes culminating in hemorrhagic fever [3, 4]. Recently, CCHFV was designated as one of ten high priority emerging infectious diseases by the World Health Organization (WHO) due to its epidemic emergence potential and lack of approved medical countermeasures [5].

CCHFV has a tripartite, negative-sense RNA genome comprising small (S), medium (M) and large (L) segments. The S segment encodes the nucleocapsid protein (N), the M segment encodes the glycoprotein open reading frame (ORF) that is cleaved into two structural glycoproteins (G_N_ and G_C_) and nonstructural proteins, and the L segment encodes the RNA-dependent RNA polymerase (RdRp; reviewed in [6]). CCHFV is the most genetically diverse arthropod-borne virus, divided into six clades, where the nucleotide sequence differences can range from 20% for the S segment, 22% for the L segment, and 31% for the M segment [7]. The overall impact of this genetic diversity on human pathogenesis is poorly understood. Infection by this virus can lead to severe disease in humans, with a global average case fatality rate (CFR) estimate of 13% [8]. However, the CCHF CFR shows a tremendous amount of variation geographically, with 5% reported in Turkey, but much higher CFRs reported in India (60%) and Tajikistan (82%) [8, 9]. While some CFR variability may be the result of underdiagnosed cases of mild CCHF [7], strain heterogeneity along with other factors, such as availability of advanced medical care, may also account for the broad CFR range.

Until recently, there was no immunocompetent laboratory animal disease model available for the study of CCHF. A cynomolgus macaque severe disease model was recently established for CCHF using an intravenous (IV) or combined IV and subcutaneous (SC) exposure with a high dose (>6 log_10_ PFU) of the European CCHFV isolate, Kosova Hoti. Non-human primates (NHPs) became viremic and developed a severe and sometimes fatal disease [10]. Several features typical of human disease, including elevated liver enzymes, thrompocytopenia, leukopenia and fever [11, 12] were present in infected NHPs. The development of the cynomolgus macaque model represents an important advancement in the field where an immunocompetent CCHF animal model is now available to study pathogenic disease mechanisms and evaluate medical countermeasures against CCHFV.

The ability of CCHFV to persist and cause long-term sequelae following infection in humans has not been well studied. Furthermore, CCHF in patients with latent or active tuberculosis has not been described despite the significant geographical overlap of these two important diseases. Tuberculosis threatens millions of lives world-wide and is the leading cause of death due to a bacterial pathogen. The WHO estimates that there were more than 10 million new active cases of tuberculosis and close to 1.3 million deaths in 2017 alone [13]. Geographically, the highest incidence of tuberculosis occurs in Asia and Africa. Only 5–10% of individuals infected with *Mycobacterium tuberculosis* (*M*. *tuberculosis*) develop active tuberculosis over their lifetime and the remaining 90–95% of infected individuals remain asymptomatic and are latently infected. Latent tuberculosis is the result of a complex set of interactions between mycobacterium and the host immune response, where the bacilli exist within granulomas and can subsequently reactivate to cause active disease [14]. *Mycobacterium bovis* (*M*. *bovis*) can also cause active or latent tuberculosis in humans, who can become infected after eating or drinking contaminated unpasteurized dairy products or by exposure during slaughter of infected animals. Tuberculosis caused by *M*. *tuberculosis* and *M*. *bovis* have similar symptoms, including fever, night sweats and weight loss [15]. Progressive granuloma formation is a hallmark of chronic mycobacterial infections. This organized structure of cells consists of a central area of necrotic debris surrounded by a layer of epithelioid macrophages, foamy macrophages, and multinucleate giant cell macrophages, further surrounded by lymphocytes, plasma cells and fibroblasts. The immune control of latent mycobacterial infections can be affected by co-infection with other pathogens. The most well-known example is the effect of the co-infection of *M*. *tuberculosis* and human immunodeficiency virus (HIV), which has been described as a syndemic (i.e. synergistic epidemic) [16]. The NHP model of tuberculosis is the “gold-standard” for modeling tuberculosis and have been used to study HIV co-infection [17].

Here we compared the disease progression in cynomolgous macaques of the previously reported European strain of CCHFV (Hoti) to that caused by an Asian strain, which was isolated from a fatal case of CCHF that occurred in a U.S. military service member serving in Afghanistan [18]. Unexpectedly, during the course of our studies, we determined that some CCHFV infected NHPs had latent tuberculosis. This finding allowed us a unique opportunity to observe disease parameters of both pathogens in the same host. Because tuberculosis and CCHF occur in overlapping geographic regions, our results have important public health implications for these emerging pathogens.

## Results

### Cynomolgus macaques infected with two diverse strains of CCHFV develop similar levels of viremia, fever, and signs of clinical disease

**Fig 1:**
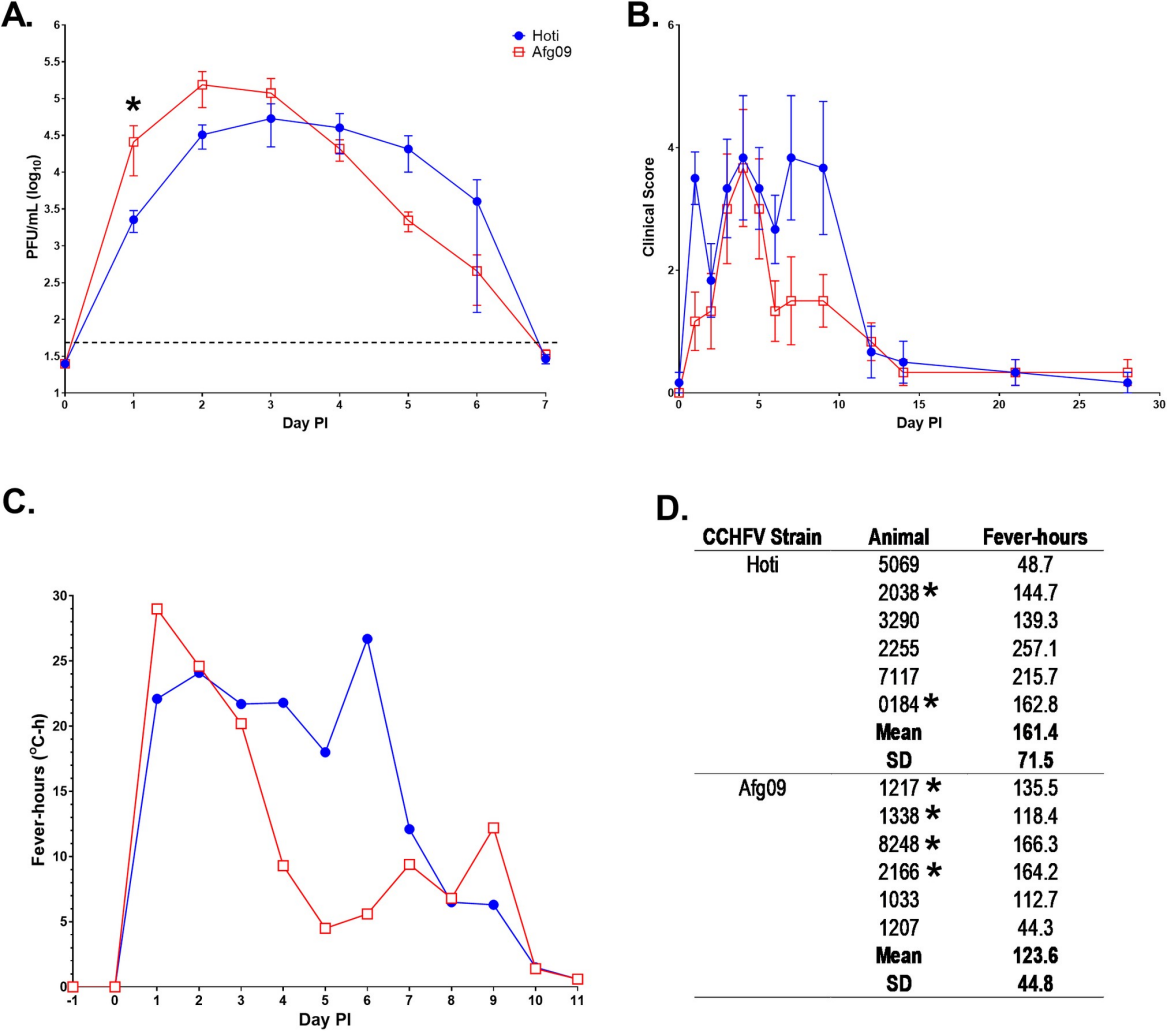
Viremia, clinical score, and fever responses in cynomolgus macaques infected IV with CCHFV strain Hoti or Afg09. A). Viremia was determined by standard plaque assay. The symbols represent the mean value and the error bars represent the standard error of the mean. The dashed line represents the assay LOD. The asterisk indicates a statistically significant difference in viremia was detected between the two groups by mixed model repeated measures ANOVA (p = 0.0045). B). Clinical scores were determined on each animal when anesthetized for blood collection. The symbols represent the mean value and the error bars represent the standard error of the mean. C). The significant temperature responses are indicated as fever-hours and shown for the animals infected with CCHFV strain Hoti or Afg09. D). The total fever-hours for each NHP with group means and SD. Asterisks indicate the NHPs that had evidence of a latent mycobacterial infection.

Cynomolgus macaques were infected IV with a high dose of CCHFV strain Kosova Hoti (6.6 log_10_ PFU; hereafter referred to as Hoti; n = 6) or strain Afg09-2990 (6.2 log_10_ PFU; hereafter referred to as Afg09; n = 6) to directly compare the pathogenesis of the two isolates. The complete genomes for these virus strains have been described previously, where the Hoti strain was found to be phylogenetically related to the Europe/Turkey group [19], and the Afg09 strain was related to Asian strains [20]. We confirmed the gene sequences of our virus stocks and compared strain Hoti and Afg09, which were found to share nucleic acid and amino acid sequence homologies across their L/M/S segments of 87%/80%/87% and 97%/85%/97%, respectively (S1 Fig). At the completion of the study, we learned that two NHPs infected with strain Hoti (animal numbers 0184, and 2038) and four NHPs infected with strain Afg09 (animal numbers 1217, 1338, 2166, 8248) had evidence of a latent mycobacterial infection with granulomas in the lung, liver, and/or lymph node (discussed in more detail in a subsequent section). Animals in both groups became viremic, peaking on day 2 post-infection for NHPs exposed to the Afg09 strain (5.2 log_10_ PFU/mL) or on day 3 post-infection for NHPs exposed to the Hoti strain (4.7 log_10_ PFU/mL; Fig 1A and S2A and S2B Fig). Overall there was no statistically significant difference (p = 0.2778) in viremia between animals infected with either strain except on day 1 post-infection (p = 0.0045). While most NHPs exhibited transient weight loss subsequent to virus exposure (S2C and S2D Fig), no animal met euthanasia criteria or otherwise succumbed to disease. Animals infected with both CCHFV strains lost a significant amount of weight compared to baseline values prior to virus exposure (ANOVA; p<0.0001), and had significant changes over time for both groups (ANOVA; p = 0.0304). However, the rate of weight change was similar between animals exposed to CCHFV strains Hoti and Afg09 (p = 0.4331).

We observed clinical signs of disease as early as day 1 post-infection, and all animals experienced clinical illness by day 3 post-infection (Fig 1B; S2G and S2H Fig), including anorexia, lymphadenopathy, fever, and some animals (n = 2/12) developed a rash. One animal infected with the Afg09 strain developed a petechial rash that appeared in the axillary region on day 2 post-infection and completely resolved by day 5 post-infection (S3A–S3D Fig). An animal infected with the Hoti strain developed a macular rash in the axillary region on day 3 post-infection that resolved by day 5 post-infection (S3E and S3F Fig). Vaginal bleeding was observed in one animal infected with the Afg09 strain on days 1 and 3 post-infection and another animal infected with the Afg09 strain on day 6 post-infection. Vaginal bleeding occurred in one animal infected with the Hoti strain on days 5–7 post-infection and in another animal infected with Hoti on day 7 post-infection. However, distinction of these vaginal bleeding events from normal estrus is difficult to establish. The only other bleeding that was observed was epistaxis on day 7 post-infection in one animal infected with the Hoti strain.

All animals developed a fever response (defined as >3 standard deviations above baseline) which was prominent from days 1 through 9 and had fully resolved by day 10 post-infection. The mean time to reach fever state was 1.09 days for animals infected with Afg09 strain and 1.03 days for animals infected with the Hoti strain. There was not a statistically significant difference in the mean time to fever state for animals infected with the Hoti vs. Afg09 strain. The fever-hours (Fig 1C and 1D and S2E and S2F Fig) is the sum of the significant temperature elevation values, which gives an indication of the intensity of the fever by measuring the area under the curve. Ten of the 12 NHPs had moderate fevers (>100 fever-hours), and one NHP in each group had mild temperature changes (<100 fever-hours; animals 5069 and 1207). Hyperthermia was more prolonged in animals exposed to the Hoti strain with mean fever-hours of 161.4 compared to 123.6 for NHPs exposed to the Afg09 strain, but this was not statistically significant.

### CCHFV-infected NHPs experienced marked aberrations in hematological and chemistry values

**Fig 2:**
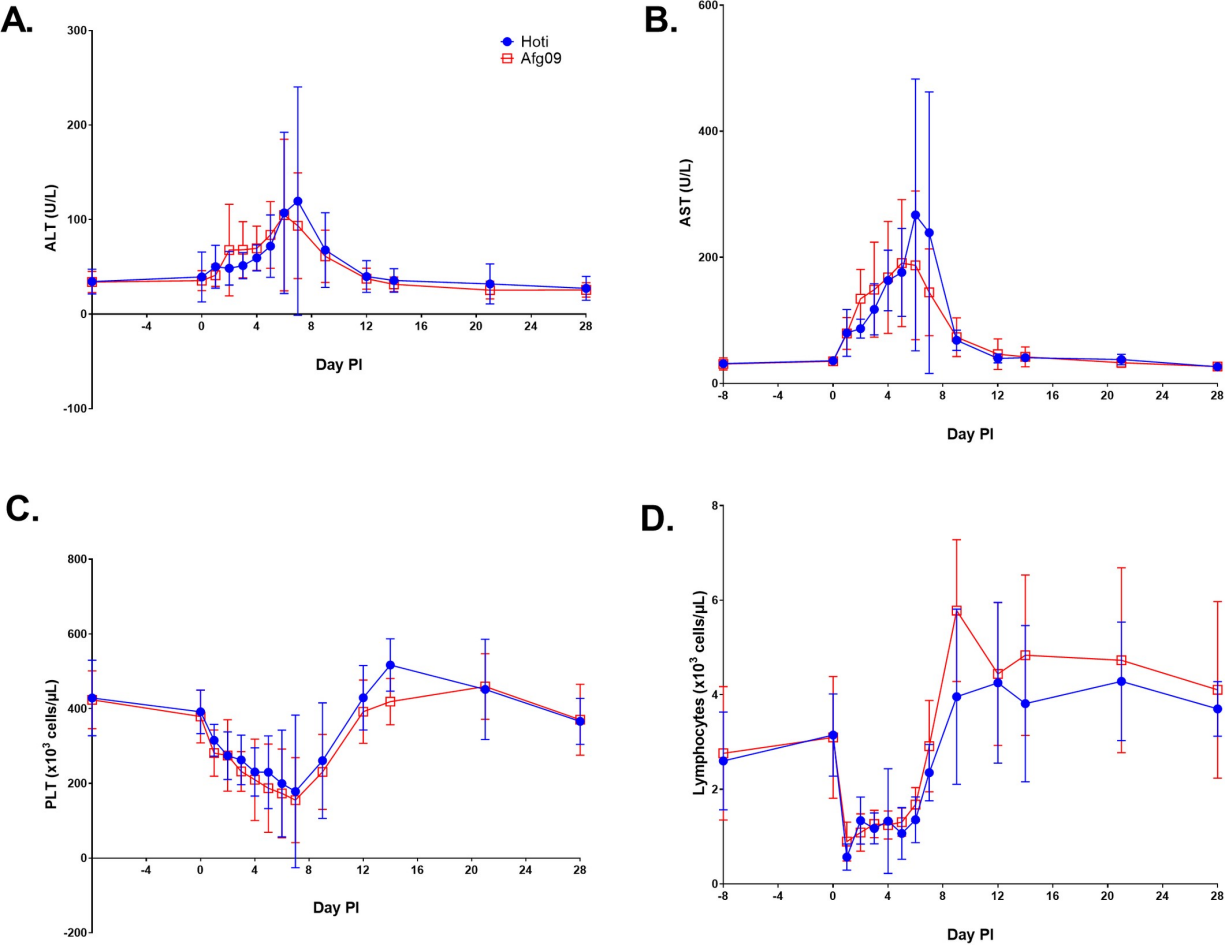
Blood chemistry and hematology results in cynomolgus macaques infected IV with CCHFV strain Hoti or Afg09. A) ALT. B) AST. C) PLT. D) Lymphocytes. The symbols represent the mean value and the error bars represent the standard error of the mean.

The blood chemistry and complete blood count (CBC) values were determined on days -8, 0–7, 9, 12, 14, 21 and 28 in NHPs exposed to the Hoti and Afg09 strains. Generally, all NHPs exhibited marked changes in multiple analytes as early as day 1 post-infection. Results from repeated measures ANOVA (S1 and S2 Tables) showed significant changes over time for all analytes except blood urea nitrogen (BUN), basophils (BASO), mean corpuscular volume (MCV), and mean platelet volume (MPV) indicating that the CCHFV disease course affects chemistry and hematology function regardless of strain. Results from repeated measures ANOVA indicated some statistically significant differences in the hematology and chemistry results among animals infected with the Afg09 or Hoti strains, but not in the interaction between strain and time. The alanine aminotransferase (ALT), aspartate aminotransferase (AST), and alkaline phosphatase (ALP) levels increased during the first 7 days post-infection, which is an indication of hepatocellular damage (Fig 2A and 2B and S4A Fig). The amylase (AMY) levels decreased during the first two days post-infection, suggesting virus was impacting kidney function (S4B Fig). Other indicators of liver disease occurring in CCHFV infected animals included a significant decrease in total protein and albumin (ALB) levels (S4C and S4D Fig). NHPs experienced thrombocytopenia, lymphopenia, and leukopenia (Fig 2C and 2D and S4E Fig) during the first 7 days post-infection. The neutrophils increased until day 3 post-infection followed by neutropenia (S4F Fig). The eosinophils and monocytes decreased slightly followed by a significant increase that peaked on day 9 post-infection (S4G and S4H Fig).

### Serum cytokine and antibody responses in CCHFV-infected NHPs

Serum cytokine levels were evaluated via a multiplexed NHP cytokine detection kit on days 3 and 6 post-infection and were assessed relative to day 0 levels (S5 Fig). We observed an increase in a number of cytokine markers including the interleukins (IL)-1RA, IL-6, IL-10, IL-15, and IL-18 as well as monocyte chemo- attractive protein (MCP)-1 and interferon (IFN)-γ. For all the cytokines listed, we observed elevated levels of the markers at both days 3 and 6 post-infection, but with a general decrease in signal from day 3 to 6. An exception to this for both strains was IL-10 and IFN- γ levels which remained constant or even increased from day 3 to 6 post-infection (S5C and S5G Fig). Overall no statistically significant differences in cytokine levels was observed for animals infected with the Hoti vs. Afg09 strain.

Host antibody response was measured by a combination of multiplexed immunoassays and neutralization assays using a virus-like particle (VLP) system. For detecting immunoglobulin reactivity against distinct viral antigens (N or G_N_), we employed a novel bead-based assay which has an enhanced sensitivity profile relative to conventional ELISA [21]. Antigen-coupled beads were probed with NHP sera and then exposed to detector antibodies for either IgM or IgG. In observing the timing of host IgM response to infection by both CCHFV strains (S6A Fig), we observed a rapid IgM response to CCHFV N, starting 3 days after infection, peaking 7 days post-infection and deteriorating rapidly thereafter. Anti-N IgM kinetics between the two strains were virtually identical. By contrast, host IgM seroconversion against recombinant G_N_ antigen was much slower for both strains, reaching peak levels several days after N before starting to diminish. Unsurprisingly, host IgG response against both antigens was slower relative to IgM (S6B Fig). Virus-neutralization response was evaluated using a VLP system with glycoproteins based on the IbAr 10200 CCHFV strain [22]. We chose IbAr 10200 VLPs as a neutral interrogator of broad neutralizing antibody response by the host against CCHFV glycoprotein complex (GPC) as it is from a clade distinct from both Hoti and Afg09, but nonetheless shares 90% and 85% protein conservation with each of these isolates’ respectively. The timing of neutralization response was assessed in pooled NHP sera from each treatment group collected at different time-points. We observed the emergence of neutralizing antibodies in sera by day 9 post-infection for both groups, with broadly similar kinetics and endpoint titers between the Hoti and Afg09 infected groups (S6B and S6C Fig). Endpoint neutralization titers (day 28–30 post-infection) were equivalent in both groups with no significant difference (S6D Fig).

### Virology and pathology findings in CCHFV-infected NHP tissues

**Fig 3:**
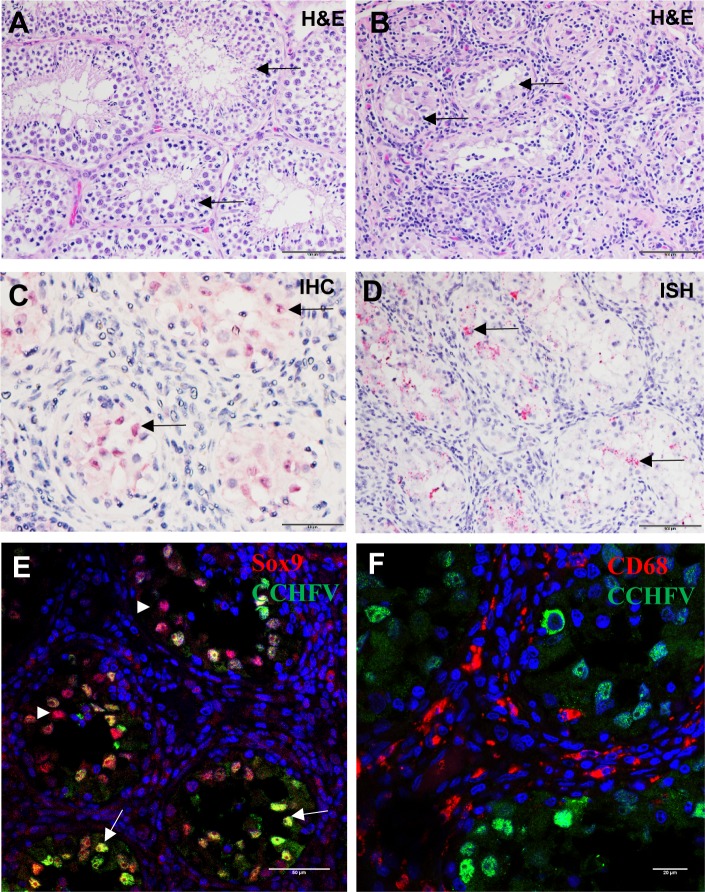
Histologic, IHC, and ISH findings in the testes of CCHFV-infected cynomolgus macaques. A). Hematoxylin and eosin staining of an animal with a normal appearing testis and no evidence of CCHFV infection. Arrow, seminiferous tubules. B). Hematoxylin and eosin staining of an animal with inflammation of the testis. Arrow, seminiferous tubules. C). IHC staining demonstrating positive (red) staining of presumed Sertoli cells in an area of inflammation in the testis of an infected animal. Arrow, CCHFV antigen-positive cells. D). ISH staining demonstrating that CCHFV RNA is detected in the luminal debris and Sertoli cells in the testis of a CCHFV-infected animal. Arrow, CCHFV RNA-positive Sertoli cells. E) IFA demonstrating that CCHFV (green) infects Sertoli cells (red). White arrowheads indicate CCHFV negative Sertoli cells labeled by an anti-Sox9 antibody while arrows indicate CCHFV positive Sertoli cells labelled by an anti-Sox9 antibody. F). IFA demonstrating that CCHFV (green) does not infect CD68^+^ macrophages/monocytes (red) in the testis.

**Fig 4:**
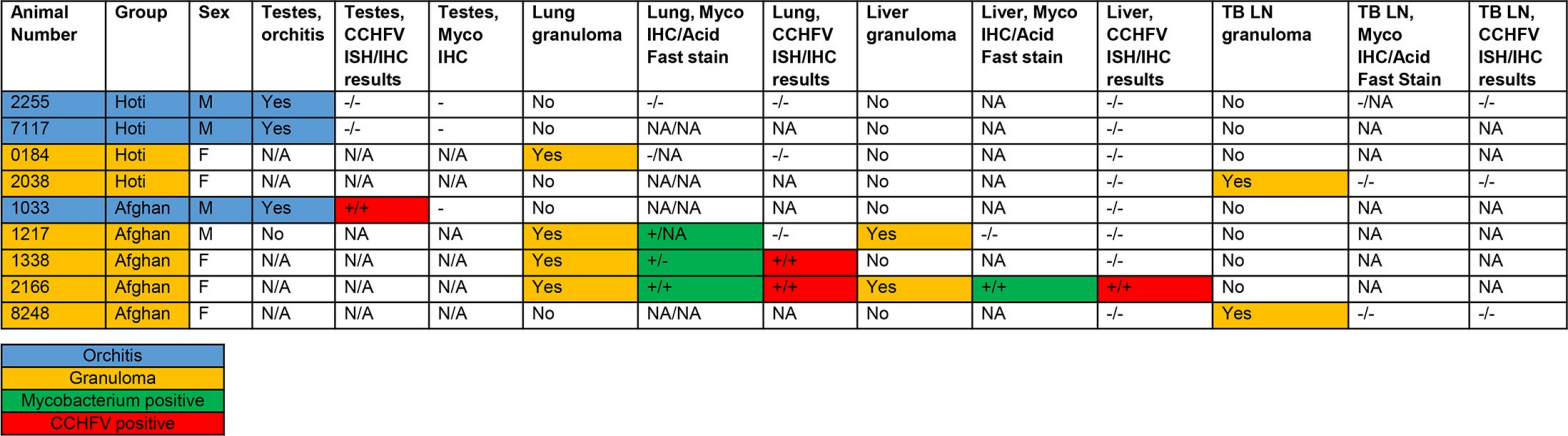
Summary of the histologic, ISH, IHC, and histochemical staining findings for select animals/tissues. Animals with orchitis are indicated by blue shading, the presence of granulomas by orange shading, mycobacterium positive tissues by green shading, and CCHFV RNA/antigen by red shading. N/A = not applicable and NA = not assessed.

All NHPs survived infection. On day 28–30, necropsy was performed on each animal and a small piece of the following tissues were collected and evaluated for virus by plaque assay: lymph node (axillary), liver, spleen, kidney, testis, epididymis, ovary, eye (optic nerve/retina), eye fluid (aqueous and vitreous humor), and brain. No viable virus was detected in any of these samples by plaque assay. All animals were clinically normal at the time of necropsy, and few animals had significant gross lesions. Animal 2166 had a focal 0.25 cm mass in the caudate lobe of the liver, and animal 1033 had a right testis that was approximately half the size of the left testis. There were no other significant gross lesions recorded at the time of necropsy. Significant histologic lesions suggestive of CCHFV infection were not observed in any tissue except the testes of three male monkeys. Animals 2255, 7117, and 1033 had unilateral inflammation in the testis (orchitis). The lesions were characterized by lymphocytic and plasmacytic inflammation, spermatogonia/germ cell loss, luminal debris, seminiferous tubule atrophy and loss, and interstitial fibrosis. Lesions in the testis of animal 1033 were multifocal and mild; those in 2255 and 7117 were focally extensive, affecting around 25–70% of examined sections (Fig 3A and 3B). Immunohistochemistry (IHC) and RNA *in situ* hybridization (ISH) performed on the testis were positive for CCHFV antigen/RNA in animal 1033, and were negative in animals 2255 and 7117 (Fig 3C and 3D; Fig 4). CCHFV RNA/antigen was detected in the seminiferous tubules, which is the site of sperm production and an immune privileged area of the body. To determine the location of the viral antigen, we performed immunofluorescence (IFA) staining using an antibody against Sox9, which is an established cell-specific marker for Sertoli cells. IFA demonstrated that CCHFV specifically infects Sertoli cells and not CD68^+^ macrophages/monocytes in the testis (Fig 3E and 3F).

**Fig 5:**
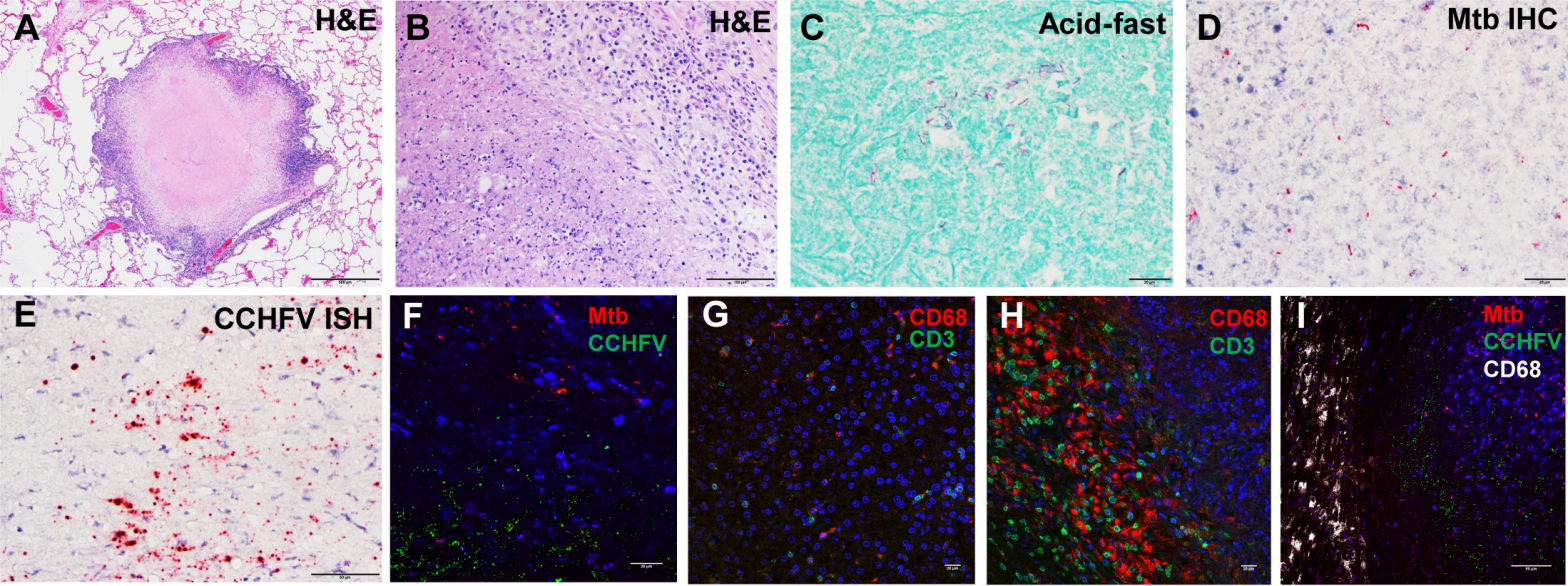
Histologic, acid-fast, IHC, ISH, and IFA findings in the tissues of CCHFV-infected cynomolgus macaques. A). Hematoxylin and eosin staining showed a granuloma in the lung. B). Hematoxylin and eosin staining of an animal with granulomatous inflammation in the liver. C). Acid-fast stain showed myriad bacilli (*Mycobacterium species*) are visible within the necrotic central area of a granuloma in the liver. D) IHC straining demonstrating that mycobacterium (red) is detected within the necrotic central area of a granuloma in the liver. E). Representative ISH staining demonstrating that CCHFV RNA (red) is detected at the periphery of the necrotic center of an animal with a lung granuloma. F). IFA demonstrating that both mycobacteria (red) and CCHFV (green) persist in a lung granuloma. G-H). Excessive number of T cell (green) and macrophages/monocytes (red) surrounds lung granuloma (H) in contrast to adjacent uninfected tissue (G). I). Neither CCHFV (green) nor mycobacteria (red) persists in CD68^+^ macrophages/monocytes (gray), which surrounds the lung granuloma.

Six animals had granulomas and/or granulomatous lesions in the lungs, tracheobronchial lymph node, and/or liver (Fig 4; Fig 5A and 5B) that were suspect for mycobacterial infection. A diagnosis of mycobacterial infection in lung and liver (2166) granulomas was confirmed in three animals (1217, 1338, 2166; Afg09 group) using acid-fast histochemical staining and mycobacterium-specific IHC (Fig 4; Fig 5C and 5D). IHC for mycobacteria was also performed on the testis of the three animals with orchitis (2255, 7117, 1033), all with negative results. CCHFV IHC and ISH was performed on all granulomatous lesions. Positive IHC and ISH was observed in lung granulomas in animals 1338 and 2166, as well as positive results in the liver granuloma in animal 2166 (Fig 4; Fig 5E). IFA demonstrated that both mycobacteria and CCHFV were present in the necrotic area of the granuloma. Compared to adjacent normal liver tissue, there was an excessive number of CD3^+^ T cells and CD68^+^ macrophages/monocytes surrounding the granuloma (Fig 5G and 5H). Interestingly, neither mycobacteria nor CCHFV infection was detected in surrounding CD68^+^ macrophages (Fig 5I). We did not collect samples for sequencing from the animals in the current study, but sampling of granulomatous lesions from other NHPs housed in the same colony were found to be positive for *M*. *bovis*.

### Overall summary of the disease progression in CCHFV-infected NHPs

**Fig 6:**
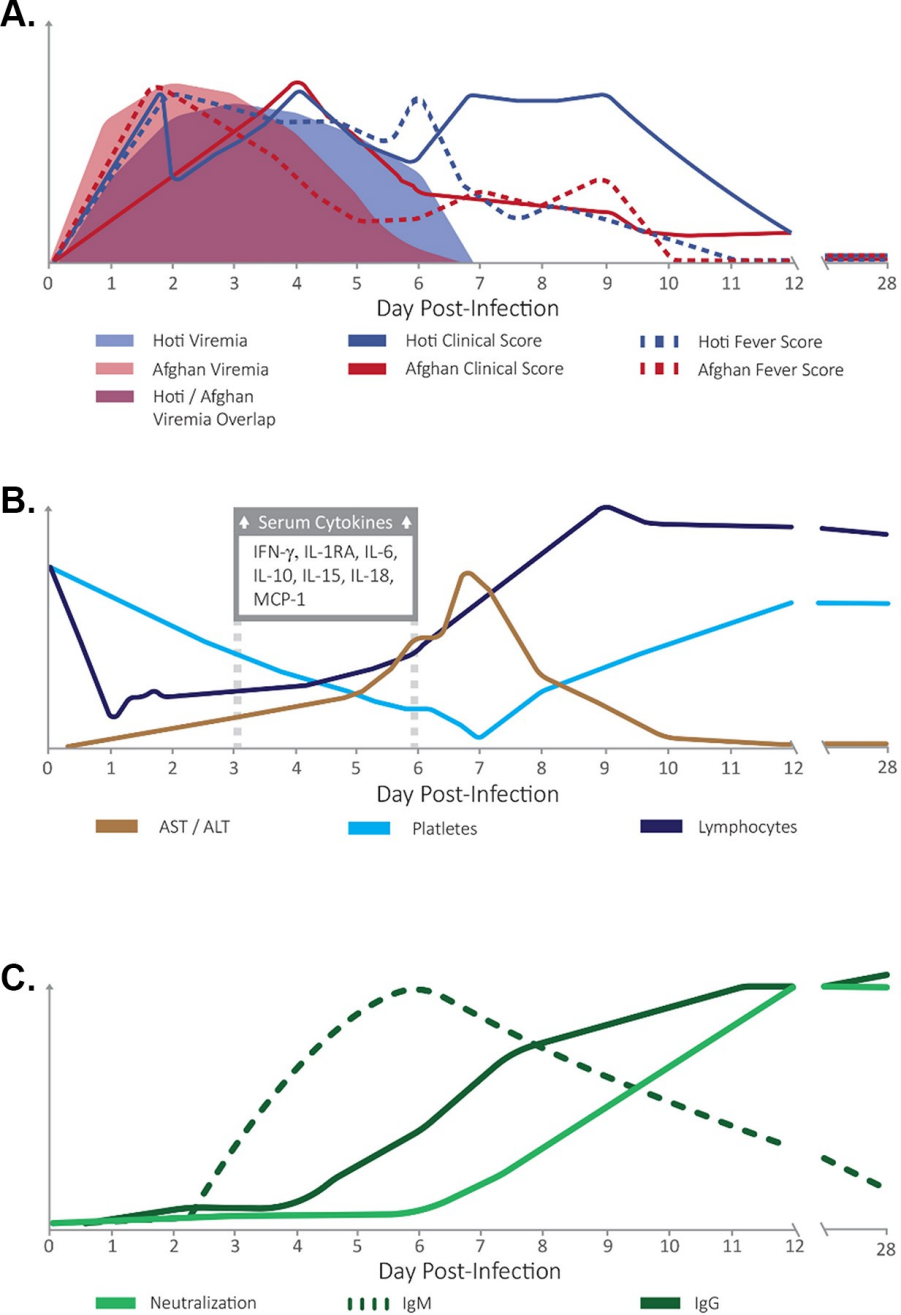
Comparative summary of the disease progression in cynomolgus macaques infected intravenously with CCHFV strain Hoti or Afg09. A) Comparison of fever and clinical score for both strains in comparison to viremia over time. B) Progression of AST/ALT, hematology, and serum cytokine levels over time. C) Development of neutralizing antibody and anti-nucleocapsid IgM and IgG in response to CCHFV infection over time. Due to their nearly identical kinetics, all parameters displayed in B) and C) are shown as merged averages between strains and represent “generic” CCHFV responses. These figures are intended to be qualitative and are only meant to convey relative trends over time.

The qualitative overview of the progression of CCHF as modeled in both virus exposure groups is depicted in Fig 6 and includes fever data, viremia, prominent changes in hematology and blood chemistry, and host markers associated with CCHF. Although we did not observe mortality, all animals demonstrated signs of clinical illness, viremia, significant changes in clinical chemistry and hematology values, serum cytokine profiles consistent with CCHF in humans, and seroconversion against CCHFV antigens. Overall, we found that the European and Asian CCHFV strains caused very similar disease profiles in cynomolgus macaques, and that the virus may persist in the testes and within lung and liver granulomas in animals concurrently infected with latent tuberculosis.

## Discussion

The development of a cynomolgus macaque disease model has provided a major advance for CCHFV research [10]. Our results are largely consistent with those from the earlier report and further demonstrate the utility of the model for another genetically diverse CCHFV strain. In contrast to the previous study, we demonstrated a febrile response in all animals that lasted on average between 5–7 days post-infection. The earlier study found that elevated temperatures were only observed on day 1 post-infection in 2 of 4 animals, which was likely due to measuring temperature changes rectally in anesthetized animals. The use of telemetric temperature monitoring in the current study clearly offers a more sensitive and accurate means to evaluate the febrile response, which is an important endpoint criteria for evaluating the effectiveness of medical countermeasures.

While we achieved a clearly discernible disease state in all animals exposed to both CCHFV strains, none approached euthanasia criteria and all NHPs (n = 12) survived challenge. In contrast, 75% (3/4) of the animals that were exposed IV with CCHFV strain Hoti in the earlier study met euthanasia criteria. The data indicate that more animals in that study experienced signs of severe disease such as body/facial edema and bleeding. Differences in scoring criteria between both studies may account for why no animals met euthanasia criteria in the current study. Other possible explanations could include variables in virus stock, dose, and genetic background of the NHPs. These differences should be further examined in an effort to refine and standardize the CCHF NHP model.

The typical progression of CCHF in humans has been described in four distinct phases: incubation (3–7 days post-infection), prehemorrhagic (4–5 days), hemorrhagic (2–3 days), and convalescence (10–20 days post-onset of symptoms) [12, 23]. Unlike the earlier NHP study with strain Hoti, we observed almost no incubation period in either of our infection groups, as evidenced by rapid fever, viremia, and lymphopenia within one day of infection. It should be noted, however, that in both studies pathogenesis was considerably faster than CCHF in humans. The difference in the incubation period between humans and NHPs could be related to the route of exposure (i.e. tick transmission/nosocomial vs. IV exposure) and viral dose.

The prehemorrhagic period in humans has been characterized by the onset of fever, headache, myalgia, and dizziness. Some cases have experienced diarrhea, nausea, vomiting, and hyperemia of the face, neck and chest (reviewed in [24]). In the current NHP study, we observed fever and in some instances diarrhea and anorexia as evidence of the prehemorrhagic period with no major differences between the two strains. The serum cytokine monocyte chemo‐attractive protein (MCP-1), which is thought to be an activator of natural killer cells and important for effective innate immunity, was elevated in both groups of NHP [25, 26]. Elevated serum MCP-1 was also reported in the earlier NHP study and has been described for human CCHF cases [10, 27]. Other markers that were upregulated during the early active infection phase included IFN-γ, IL1-RA, IL-6, IL-10, IL-15, and IL-18; all of which have been reported in cases of human disease [24, 28].

Hallmarks of the hemorrhagic period in humans include the development of petechiae to large hematomas on the skin or mucous membranes. The nose, gastrointestinal system, uterus and urinary tract, and respiratory tract are common sites where bleeding has occurred [24, 29]. Less commonly, bleeding has been described in the vagina, gums, and cerebrum [30]. Similarly, in our study, we observed signs of rash, vaginal bleeding, and epistaxis in a few cases with no major differences between the two strains.

Humans have been described to enter the convalescence period 10–20 days after the onset of illness [24], which is similar to what we observed in the NHP model. We did not observe significant differences in the kinetics of host IgM, IgG, or neutralizing responses between virus strains. In addition, we report here the first ever temporal resolution of host primate IgM response to CCHFV nucleocapsid antigen, a viral marker frequently used in diagnostic immunoassays (ELISAs, etc.) [31, 32]. The rapid host IgM response we observed and its subsequent degradation were indistinguishable between these distantly-related viral strains, which reinforces the utility of this viral marker as a immunodiagnostic target of both acute, and early convalescent CCHFV infection [33].

The persistence of CCHFV in human survivors has not been described. Possible human sexual transmission of CCHFV has been reported in only a few cases [34, 35]. One example described a possible case of the sexual transmission of CCHFV by a man in the convalescent phase of disease [34]. Another report suggested sexual transmission of CCHFV among spouses in the southern regions of Russia [35]. These spouses had sexual contact with the index cases at the end of the incubation period or during the early stage of a mild form of CCHF with no hemorrhagic symptoms in the first infected spouse. However, all of these cases only suggest probable sexual transmission of CCHFV as investigators were unable to isolate virus in the seminal fluid and could not completely rule out possible CCHFV transmission to the partner by a viremic animal or through a tick bite. More convincing evidence for sexual transmission of CCHFV came from a case report in which a male CCHF patient with epididymo-orchitis appears to have transmitted the virus to his partner. This observation suggested that the virus could replicate in the male genital tract [36]. Our study provides the first direct evidence that CCHFV can replicate in the male genital tract where we observed orchitis in 50% (3/6) of male NHPs infected with CCHFV. CCHFV viral RNA and antigen were detected by ISH/IHC in the testes of one of these animals suggesting that most of the animals (2/3) had already cleared CCHFV infection, but still had residual testicular damage by day 28 post-infection. Interestingly, histologic lesions were more extensive in the animals where no CCHFV RNA or antigen was detected, suggesting that the inflammatory response may have cleared the virus from the testes but left severe tissue damage. The single animal that was positive for CCHFV RNA/antigen had a testis that was approximately half the size of the other testis indicating testicular atrophy. This observation of gross testicular atrophy along with the histologic lesions indicating damage such as spermatogonia/germ cell loss and seminiferous tubule atrophy and loss suggest a deleterious effect on the overall reproductive function of the testes in these 3 NHPs. Furthermore, IFA demonstrated that CCHFV specifically infects Sertoli cells, which are essential for spermatogenesis. Sertoli cells have also been reported as a target of Zika virus (ZIKV) in immunodeficient mice and a cellular reservoir of persistent infection of Marburg virus [37]. Future studies with CCHFV in NHPs and human cases should attempt to isolate RNA and infectious virus from the semen.

It is well documented that other viruses that cause hemorrhagic fever such as Ebola [38, 39], Marburg [40, 41], and Lassa [42] can be sexually transmitted in semen. Our results and the aforementioned case reports suggest that CCHFV is another virus that causes hemorrhagic fever that can be sexually transmitted. Collectively, these findings have important implications for nonvector-borne vertical transmission, as well as long-term potential reproductive deficiencies in CCHFV-infected males.

It is also possible that CCHFV might persist in other immune-privileged sites similar to what has been described for Ebola virus (EBOV) and other re-emerging viruses such as ZIKV. However, we did not detect CCHFV in ocular tissues or the central nervous system. The only other location where we detected persistent CCHFV is within the granulomas of animals with concurrent mycobacterial infection. CCHFV RNA and antigen were detected within the necrotic region and not the CD68^+^ macrophages/monocytes, which surrounds the granuloma. It is possible that CCHFV infected circulating monocytes and/or infected tissue macrophages were recruited to the sites of inflammation to participate in the inflammatory response and became entrapped within the granulomas. Local factors influencing the immune response within the granulomas may have prevented systemic re-introduction of virus while preventing clearance of the virus within the granulomatous milieu.

Concurrent infection of CCHFV with mycobacterial infection has never been reported. These two pathogens share a significant amount of geographical overlap and our results suggest that co-infection is possible, and is probably more likely as human case numbers increase. For example, it was not until the unprecedented EBOV outbreak occurred in late 2013 resulting in more than 28,000 human infections that we began to recognize the high frequency of viral RNA persistence in immune-privileged sites or fluids [43]. The ability for viral RNA to persist has important public health implications not only due to the need to ensure the full recovery of survivors but also to decrease the risk of outbreak re-ignition caused by viral spread from apparently healthy survivors to naïve individuals. The public health burden of persistent RNA virus infection is most well recognized with HIV. *M*. *tuberculosis* and HIV co-infection in the host potentiate one another by accelerating the deterioration of immunological functions. Although the NHPs in this study co-infected with CCHFV and *M*. *bovis* did not succumb from acute disease, it is unknown what long-term sequela this co-infection might cause. We also do not know what effect this co-infection has on acute CCHF. No mortality was observed in this current study as opposed to the previously described study by Haddock et al [10]. The lack of mortality in our study does not seem to be related to latent mycobacterial infections, as animals with latent tuberculosis did not respond differently to CCHFV infection compared to those without latent tuberculosis. Future efforts at understanding the interaction between mycobacterial and CCHFV infection in NHP models will likely be very difficult to execute due to husbandry concerns in BSL-4 facilities. However, this interaction could be further explored using existing murine models as well as human clinical studies in regions of the world where both diseases are endemic.

In summary, we compared the disease progression of two diverse strains of CCHFV in the newly described cynomolgus macaque model. Both the Hoti and Afg09 strains recapitulated many of the clinical features seen in human disease and those initially reported in the strain Hoti NHP model [10]. While both Hoti and Afg09 demonstrated a broadly similar disease course, we observed a more persistent viremia, higher fever, and longer window of elevated clinical scores in the Hoti-infected NHPs relative to the Afg09 group. It will be of considerable interest for future studies to examine additional strains of CCHFV, and ascertain how variable their disease profiles are in animal models. Furthermore, this model can be used to understand mechanisms of viral RNA persistence and its effect on the development of long-term sequela, including its interactions with other pathogens such as mycobacteria. The utility of the cynomolgus macaque model of CCHF will advance the development and evaluation of medical countermeasures against this emerging infectious disease.

## Methods

### Ethics statement

This work was supported by an approved USAMRIID Institutional Animal Care and Use Committee (IACUC) animal research protocol in compliance with the Animal Welfare Act, PHS Policy, and other Federal statutes and regulations relating to animals and experiments involving animals. The facility where this research was conducted is accredited by the Association for Assessment and Accreditation of Laboratory Animal Care, International and adheres to principles stated in the Guide for the Care and Use of Laboratory Animals, National Research Council, 2011. Approved USAMRIID animal research protocols undergo an annual review every year. Animals are cared for by a large staff of highly qualified veterinarians, veterinary technicians, and animal caretakers. All personnel caring for and working with animals at USAMRIID have substantial training to ensure only the highest quality animal care and use. All steps were taken to enrich the welfare and to avoid the suffering of the animals in accordance with the “Weatherall report for the use of nonhuman primates” recommendations. Animals were housed in adjoining individual primate cages allowing social interactions, under controlled conditions of humidity, temperature, and light (12-hour light/12-hour dark cycles). Food and water were available ad libitum. Animals were monitored and fed commercial monkey chow, treats and fruit twice daily by trained personnel. Environmental enrichment consisted of commercial toys. Highly trained personnel completed all procedures under the oversight of an attending veterinarian and all invasive clinical procedures were performed while animals were anesthetized. NHPs were humanely euthanized by administration of greater than or equal to 6mg/kg Telazol until a surgical plane of anesthesia was achieved, terminally bled intracardiacly (IC), and administered 0.3–0.4ml/kg pentobarbital-based euthanasia solution (Fatal-Plus) IC.

### Virus strains, animals, and study design

CCHFV strain Kosova Hoti (Hoti) was originally isolated from the blood of a female fatal case during the epidemic in Kosovo in 2001 [19]. CCHFV strain Hoti was kindly provided by Dr. Tatjana Avšič - Županc (University of Ljubljana) and Dr. Heinz Feldmann and had been passaged 7 times in Vero E6 cells and 3 times in SW13 cells prior to receipt at USAMRIID [10]. CCHFV strain Afg09-2990 was derived from a fatal human case in a U.S. soldier in Afghanistan in 2009 [18]. It was obtained from the Bernhard Nocht Institute (Hamburg, Germany), where it had been passaged a total of 3 times in Vero E6 cells since isolation. Both strains were amplified by one passage in Huh7 cells (human liver cell line) at USAMRIID prior to use in the current study. Huh7 cells were used because CCHFV is hepatotropic and these cells produce less interferon than many immortalized cell lines. However, CCHFV does not plaque on Huh7 cells, so titers of both stocks were determined by plaque assay on SW13 cells (described below) and confirmed negative for mycoplasma and endotoxin.

Twelve healthy adult cynomologous macaques, 4 to 9 years of age, ranging in body weight from 3.4 to 7.9 kg were obtained from World Wide Primates. None of these NHPs were exposed to infectious pathogens in previous studies, and all were determined to be naïve for previous CCHFV exposure based on both VLP neutralization assay as well as an anti-CCHFV N IgG ELISA [44]. Telemetry transmitters (ITS T2J-1-Y, Konigsberg Instruments, Inc., Pasadena, CA) were surgically implanted into all animals to monitor body temperatures. Animals were allowed to recover 28 days prior to virus exposure. Prior to virus exposure (day -5) and until 28 days after CCHFV infection, body temperatures were recorded continuously using the Notocord-hem Evolution software platform (Version 4.3.0.47, Notocord Inc., Newark, NJ). Temperature values from the telemetry data files were extracted into MS Excel workbooks as 30-min averages. Additionally, 60 min temperature averages were provided to study personnel during the in-life portion of the study to support assessment of euthanasia criteria.

For each animal, telemetry data collected for 5 days prior to challenge was used as baseline data to provide the average and SD for each 30-min daily time period of a 24-hr day. Telemetry data collected during the challenge study period was compared against the correspondent baseline values and used as study telemetry data. Significant temperature elevations during the study, represented by temperature data outside +3 standard deviations of baseline values, were used to compute fever duration (number of hours of significant temperature elevation) and fever-hours (sum of the significant temperature elevations).

For the study design, the twelve animals were randomly divided into two groups (n = 6/group) where six animals per virus strain received a target dose of 6 log_10_ PFU of CCHFV (actual dose was 6.6 log_10_ PFU of strain Hoti and 6.2 log_10_ PFU of strain Afg09) diluted in 1 ml phosphate buffered saline (PBS) by IV exposure in the forearm. After CCHFV exposure, all animals were monitored for temperature changes by telemetry, weight loss, survival, and clinical signs, and blood samples were collected on days -8, 0–7, 9, 12, 14, and once a week thereafter for virological, hematological, immunological, and chemical analyses. Individual clinical sign scores ranged on a scale of 0–3 and included monitoring of responsiveness, biscuit/fruit consumption, condition of stool, temperature change from baseline, presence/absence of a rash, bleeding, lymphadenopathy, and dehydration. A cumulative score of greater than or equal to 10 would have reached euthanasia criteria. All animals were humanely euthanized 28 days after challenge, and tissues were collected for determination of viral titer and histopathology.

### Histopathology

Full necropsies and histological examination were performed by a board-certified veterinary pathologist. The following tissues were collected during necropsy: axillary, inguinal, submandibular, mesenteric and tracheobronchial lymph node; submandibular salivary gland; haired skin; brachial plexus; sciatic nerve; skeletal muscle; bone marrow (femur); eyes; brain; pituitary gland; spleen; adrenal gland; kidney; liver; stomach; duodenum; pancreas; jejunum; ileum; cecum; colon; testis/ovary; prostate gland/uterus; urinary bladder; tongue; tonsil; trachea; esophagus; thyroid gland; lung; thymus; and heart. All collected tissues were immersion-fixed in 10% neutral buffered formalin for at least 30 days. The tissues were trimmed and processed according to standard protocol [45]. Histology sections were cut at 5 to 6 μm on a rotary microtome, mounted on glass slides, and stained with hemotoxylin and eosin. Slides prepared from select tissues were stained using the American Master Tech brand Acid-Fast Bacteria Stain Kit according to the manufacturer’s instructions contained in the product insert. Briefly, tissue was deparaffinized, rinsed with alcohol, rinsed with water, immersed in carbol fuchsin followed by immersion in 1% acid alcohol followed by immersion in light green counterstain. The slide was then dehydrated with three successive alcohol changes, cleared using xylene then coverslipped.

For immunohistochemical analysis, red chromogen immunohistochemistry was performed using the UltraVision Quanto Detection System (Thermo Scientific) according to the manufacturer’s instructions. Briefly, after formalin-fixed paraffin embedded (FFPE) tissue sections were deparaffinized using xylene and a series of ethanol washes, the sections were heated in citrate buffer (pH 6.0) for 15 min to reverse formaldehyde crosslinks. After rinses with PBS (pH 7.4), the sections were blocked with CAS-Block (Life technology) containing 5% normal goat serum overnight at 4°C. Then the sections were incubated with rabbit anti-CCHFV N protein (IBT, 1:2500), rabbit anti-mycobacterium antibody (Abcam, ab20832, 1:2500), or rabbit anti-mycobacterium tuberculosis Ag85B antibody (Abcam, ab43019, 1:500) for 1 hr at room temperature. After rinses with PBS, the sections were incubated with alkaline phosphatase (AP)-conjugated polymer at room temperature for 30 min and then incubated with a Fast Red substrate solution for 12 min at room temperature. Sections were then stained with hematoxylin, air-dried, and mounted.

To detect CCHFV genomic RNA, ISH was performed using the RNAscope 2.5 HD RED kit (Advanced Cell Diagnostics) according to the manufacturer’s instructions. Briefly, an ISH probe targeting the fragment 631–2702 of CCHFV genome with GenBank accession number HM452306.1 was designed and synthesized by Advanced Cell Diagnostics. Tissue sections were deparaffinized with xylene, underwent a series of ethanol washes and peroxidase blocking, and were then heated in kit-provided antigen retrieval buffer and then digested by kit-provided proteinase. Sections were exposed to ISH target probe pairs and incubated at 40°C in a hybridization oven for 2 h. After rinsing, ISH signal was amplified using kit-provided Pre-amplifier and Amplifier conjugated to alkaline phosphatase and incubated with a Fast Red substrate solution for 10 min at room temperature. Sections were then stained with hematoxylin, air-dried, and mounted.

For immunofluorescence staining, slides were deparaffinized and treated with 0.1% Sudan Black B to reduce autofluorescence, and then tissues were heated in citrate buffer, pH 6.0 (Sigma-Aldrich), for 15 min to reverse formaldehyde cross-links. After rinses with PBS, sections were blocked overnight with PBS containing 5% normal goat serum (Sigma-Aldrich) at 4°C. Sections were then incubated with the following primary antibodies for 2 hrs at room temperature: rabbit anti-CCHFV N protein antibody (IBT, 1:2500), guinea pig anti-mycobacterium antibody (GeneTex, 1:500), rabbit anti-mycobacterium antibody (Abcam, ab20832, 1:2500), rabbit anti-CD3 antibody (Dako Agilent Pathology Solutions, 1:200), and/or mouse anti-human CD68 antibody (Dako Agilent Pathology Solutions, 1:200). After rinsing in PBS, sections were incubated with secondary goat IgG Alexa Fluor 488-conjugated anti-rabbit antibodies and with goat IgG Alexa Fluor 561 or 647-conjugated anti-mouse, anti-rabbit, or anti-guinea pig antibodies (Life Technologies, Carlsbad, CA) for 1 hr at room temperature. Sections were cover-slipped using VECTASHIELD antifade mounting medium with DAPI (Vector Laboratories, Burlingame, CA, USA). Images were captured on an LSM 880 Confocal Microscope (Zeiss) and processed using open-source ImageJ software (National Institutes of Health, Bethesda).

### Hematology, blood chemistries, and virological assays

Whole blood was added to EDTA tubes for CBC determinations using a Hemavet hematological analyzer (Drew Scientific, Dallas, TX) according to manufacturer’s instructions. Serum was isolated using a gel-based serum separator (Sarstedt, Numbrecht, Germany) and was stored at -80°C for subsequent analysis. Blood chemistries were performed on serum with a Piccolo chemistry analyzer (Abaxis, Union City, CA) utilizing General Chemistry 13 detection discs according to manufacturer’s instructions.

Serum viremia was determined by plaque assay on confluent monolayers of SW13 cells in 6 well plates. Briefly, 10-fold serial dilutions of sera were made in media and incubated on cells for 1 hr at 37°C/5% CO_2_ prior to the addition of a 1:1 mixture of 1% Seakem agarose and 2X Basal Medium Eagle with Earle's Salts (EBME) solution containing 2X EBME, 10% heat inactivated fetal bovine serum (FBS-HI) and 2% L-Glutamine. After solidification of the overlay, cells were then incubated for 48 hrs at 37°C/5% CO_2_ prior to the addition PBS with 5% Neutral Red (Gibco) for 2 hrs before plaque counting.

### Serum chemokine analysis

Sera collected on days 0, 3, and 6 of study were analyzed using a MilliPlex NHP 23-plex chemokine array (Millipore). Briefly, NHP sera was diluted 1:2 in assay buffer and was incubated on detection bead set along with reference standards for 20 hrs at 4°C. The analysis plate was subsequently processed according to manufacturer’s instructions. The samples were analyzed using a MAGPIX Analyzer (Luminex Inc., Austin, TX, USA).

### CCHFV VLP production and neutralization assay

CCHFV VLPs based on the IbAr 10200 strain were produced and purified as previously described with the following modifications [22, 27, 44]. Supernatants from transfected cells were harvested at 48, 72, and 96 hrs post-transfection. After clarification, pooled supernatant was clarified through a 0.45 μm filter. Supernatant was concentrated through an Amicon centrifugal concentrator unit with a 100 kDa cut-off (Millipore). Concentrate was then diluted 1:10 with virus resuspension buffer (VRB) before being pelleted through 20% sucrose. Final viral pellets were resuspended in 1/1000 volume of VRB relative to starting volume. VLP titer was assessed based on 50% tissue culture infectious dose (TCID_50_) assay as previously described [22].

For the neutralization assay, day 28 sera was diluted in half-log increments prior to being mixed with CCHF VLPs. Samples were incubated for 1 hr at 37°C prior to being added to SW13 target cells and incubated for an additional 24 hrs at 37°C/5% CO_2_. Data collection and 80% CCHF VLP neutralization titers were determined as previously described [22]. Sera from a human convalescent patient was used as a positive control for the assay. This system has previously been used for the evaluation of DNA vaccines in mice as well as performance of potential therapeutic monoclonal antibodies, and compares favorably with conventional PRNT assays [44].

### Serology

Recombinant CCHFV N, produced in a baculovirus expression system as previously described (13), and G_N_ (Native Antigen, Inc.) were conjugated to magnetic microspheres using the Luminex xMAP antibody coupling kit (Luminex) according to the manufacturer’s instructions. Both CCHFV antigens were based on the IbAr 10200 isolate. Briefly, 100 μL of Magplex microspheres (12.5 × 10^6^ microspheres/mL) were washed three times using a magnetic microcentrifuge tube holder and resuspended with 480 μL of activation buffer. Then, 10 μL of both sulfo-NHS and EDC solutions were added to the resuspended microspheres. The tube was covered with aluminum foil and placed on a benchtop rotating mixer for 20 min. After surface activation with EDC, the microspheres were washed three times with activation buffer prior to adding either NP or G_N_ antigen at a final concentration of 4 μg antigen/1 × 10^6^ microspheres. The tube was again covered with aluminum foil and placed on a benchtop rotating mixer for 2 hr. After this coupling step, the microspheres were washed three times with wash buffer and resuspended in 100μL of wash buffer for further use. NP and G_N_ were coupled to Magplex microsphere regions #78 and #35 (Luminex), respectively, in order to facilitate multiplexing experiments. Beads were stored at 4°C until further use.

Antigen coupled beads were mixed at a 1:1 ratio and were diluted in PBS with 0.02% Tween-20 (PBST) to 5 × 10^4^ microspheres/mL and added to the wells of a Costar polystyrene 96-well plate at 50 μL per well (2500 microspheres of each antigen bead set/well). The plate was placed on a Luminex plate magnet, covered with foil, and microspheres were allowed to collect for 60 sec. While still attached to the magnet, the buffer was removed from the plate by shaking. Then, 50 μL of 1:100 diluted serum was added to appropriate wells and the plate was covered and incubated with shaking for 1 hr at room temperature (RT). The plate was washed three times with 100 μL of PBST using the plate magnet to retain the Magplex microspheres in the wells and then 50 μL of a 1:100 dilution of goat anti-human IgM phycoerythrin conjugate (Invitrogen) or goat anti-human IgG phycoerythrin conjugate (Sigma) in PBST was added to the wells. The plate was covered and incubated with shaking for 1 hr at RT. After incubation, the plate was washed three times and the Magplex microspheres were resuspended in 100 μL of PBST for analysis on the M.AGPIX instrument. Data was evaluated as signal to noise, with noise being the average median fluorescence intensity (MFI) of each bead set in response to naïve sera.

### Phylogenetic tree analysis

CCHFV nucleic acid sequences were identified in GenBank (access date 18 April 2018). Only sequences containing at least 75% of the each segment’s length were analyzed using CLC Genomics Workbench v. 10.1.2. Amino acid sequences for the RdRp, the glycoprotein complex (GPC), and the nucleoprotein (N) were generated from these sequences. Both the nucleic acid and amino acid sequences were aligned, and the relatedness of the different CCHFV isolates are shown with phylogenetic trees generated using the following settings: Neighbor-Joining with Jukes-Cantor distance measurement and 1000 bootstrap replicates.

### Statistical analysis

SAS version 9.1.3 (SAS Institute, Inc., Cary, NC) was used to determine differences in the telemetry data mean time to fever onset by t-test and the mean viremia, weights, blood chemistry, hematology, cytokines, and antibody response by repeated measures mixed-model ANOVA. The day after challenge was used as a repeated time effect and baseline values as a time-independent covariate. Baseline values used were an average of each animals Day -8 and Day 0 values for each parameter.

## Supporting information

S1 Fig Nucleic and amino acid sequence analysis for CCHFV isolates. A) Nucleic acid sequences from the CCHFV isolates were analyzed for homology among the L, M, and S segments. B) Amino acid sequences from the CCHFV isolates were analyzed for homology for the RdRp, GPC, and NP proteins. Isolates of interest (bolded) include Kosovo Hoti, IbAr 10200, and Afg09. Selected bootstrap values are shown, and geographic locations of isolates are indicated when almost all of the isolates in that cluster are from the same region.
(TIF)

S2 Fig Individual viremia, weight, and fever responses in cynomolgus macaques infected intravenously with CCHFV strain Hoti or Afg09. Animals with granulomas detected in the lung, liver, or lymph node are indicated with an asterisk. Animals with orchitis are indicated with two asterisks. A-B) Viremia was determined by standard plaque assay. The dashed line represents the assay LOD. C-D) Weight loss for individual animals shown as percent change in baseline prior to infection. Overall animals infected with both CCHFV strains lost a significant amount of weight compared to baseline values prior to virus exposure (ANOVA; p<0.0001) and there were significant changes over time for both groups (ANOVA; p = 0.0304). E-F) The significant temperature responses are indicated as fever-hours and shown for the individual animals. G-H) Clinical scores were determined on each animal when anesthetized for blood collection.
(TIF)

S3 Fig Dermatological reactions to acute CCHFV infection. A-D) Development and resolution of petechial rash over a 3 day time period in an NHP infected with Afg09 CCHFV. E-F) Representative images of the macule rash in an NHP infected with Hoti strain CCHFV. Date of image collection is indicated. All photos depict the Axillary region.
(TIF)

S4 Fig Blood chemistry and hematology results in cynomolgus macaques infected intravenously with CCHFV strain Hoti or Afg09. A) ALP. B) AMY. C) Total Protein. D) ALB. E) WBC. F). Neutrophils. G). Eosinophils. H). Monocytes. The symbols represent the mean value and the error bars represent the standard error of the mean.
(TIF)

S5 Fig Serum Cytokine Responses in cynomolgus macaques infected intravenously with CCHFV strain Hoti or Afg09. A-G) Day 3 and Day 6 cytokine responses to CCHFV challenge with highest responding cytokines depicted. Serum samples were run in duplicate for each time point. Individual NHP cytokine responses are shown for each time point. Signal values were derived from MFI and depict fold change relative to day 0 for each NHP tested. Geometric means and standard deviations for each group time point are shown along with standard deviation bars.
(TIF)

S6 Fig Antibody responses in cynomolgus macaques infected intravenously with CCHFV strain Hoti or Afg09. A) IgM and B) IgG responses to N protein and G_N_ protein during acute and convalescent phases of CCHFV infection. All data in A and B represents individual samples run in duplicate and measured for MFI. Signal to noise values were then averaged across the entire group (Hoti or Afg09). C) Time course of neutralizing response of pooled group seras against IbAr 10200 strain CCHF VLPs. D) Individual terminal (Day 28) neutralizing responses of all NHPs challenged in this study, as assessed by inhibition of VLP infection. Pooled naïve NHP sera was used as a positive control while sera from a convalescent human sera was used as a positive control. All neutralizing titers were derived from dilution series run in duplicate. Error bars represent standard deviation for all data sets.
(TIF)

S1 Table Repeated measures ANOVA results for chemistry values. (DOCX)

S2 Table Repeated measures ANOVA results for hematology values. (DOCX)
