# Supplementary figures and images for: Persistent Crimean-Congo hemorrhagic fever virus infection in the testes and within granulomas of non-human primates with latent tuberculosis
Source: PLoS Pathog. 2019 Sep 26;15(9):e1008050. doi: 10.1371/journal.ppat.1008050 (PMC6782109; doi:10.1371/journal.ppat.1008050)

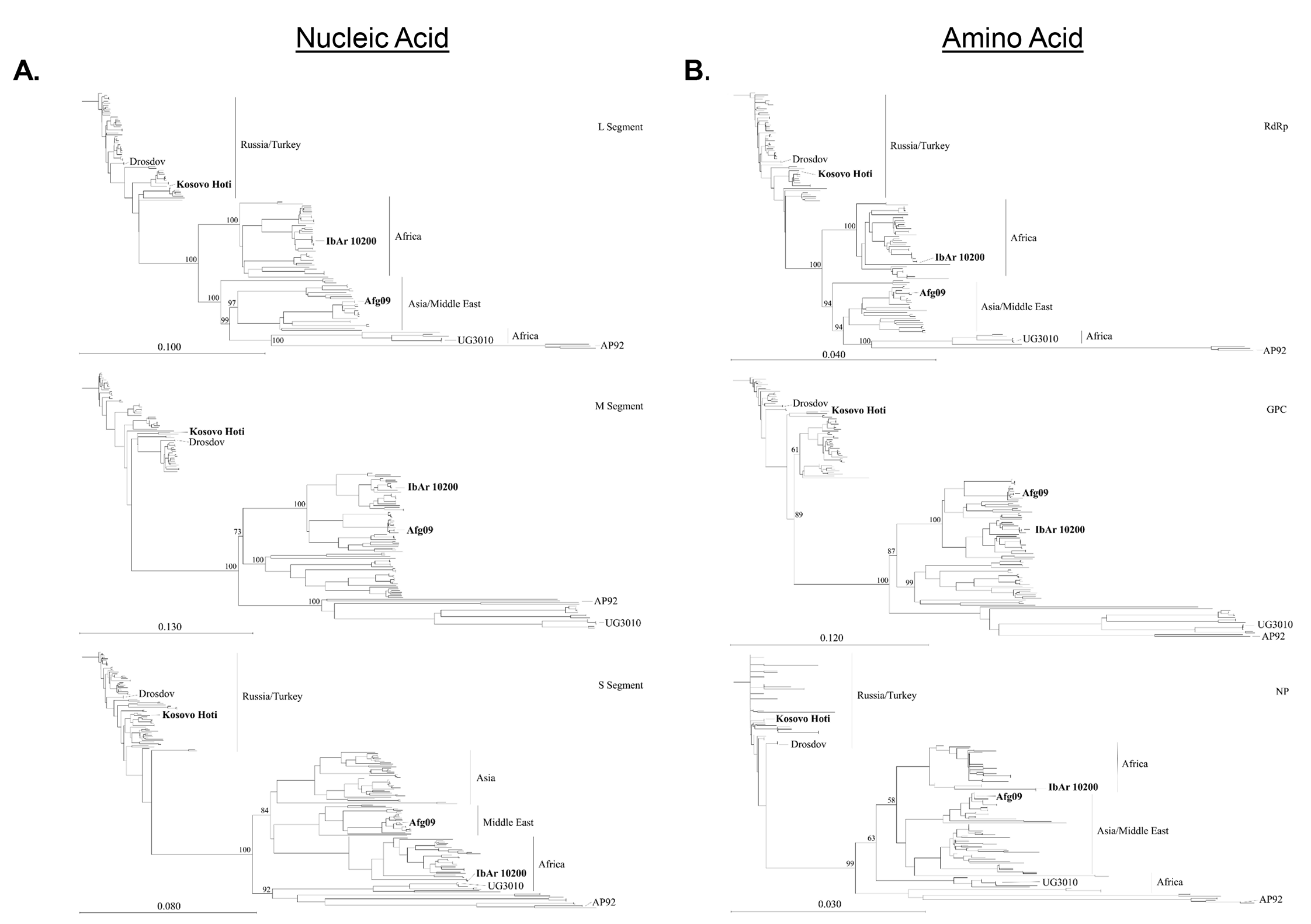

Supplement: S1 Fig — A) Nucleic acid sequences from the CCHFV isolates were analyzed for homology among the L, M, and S segments. B) Amino acid sequences from the CCHFV isolates were analyzed for homology for the RdRp, GPC, and NP proteins. Isolates of interest (bolded) include Kosovo Hoti, IbAr 10200, and Afg09. Selected bootstrap values are shown, and geographic locations of isolates are indicated when almost all of the isolates in that cluster are from the same region. (TIF) [file ppat.1008050.s001.tif]

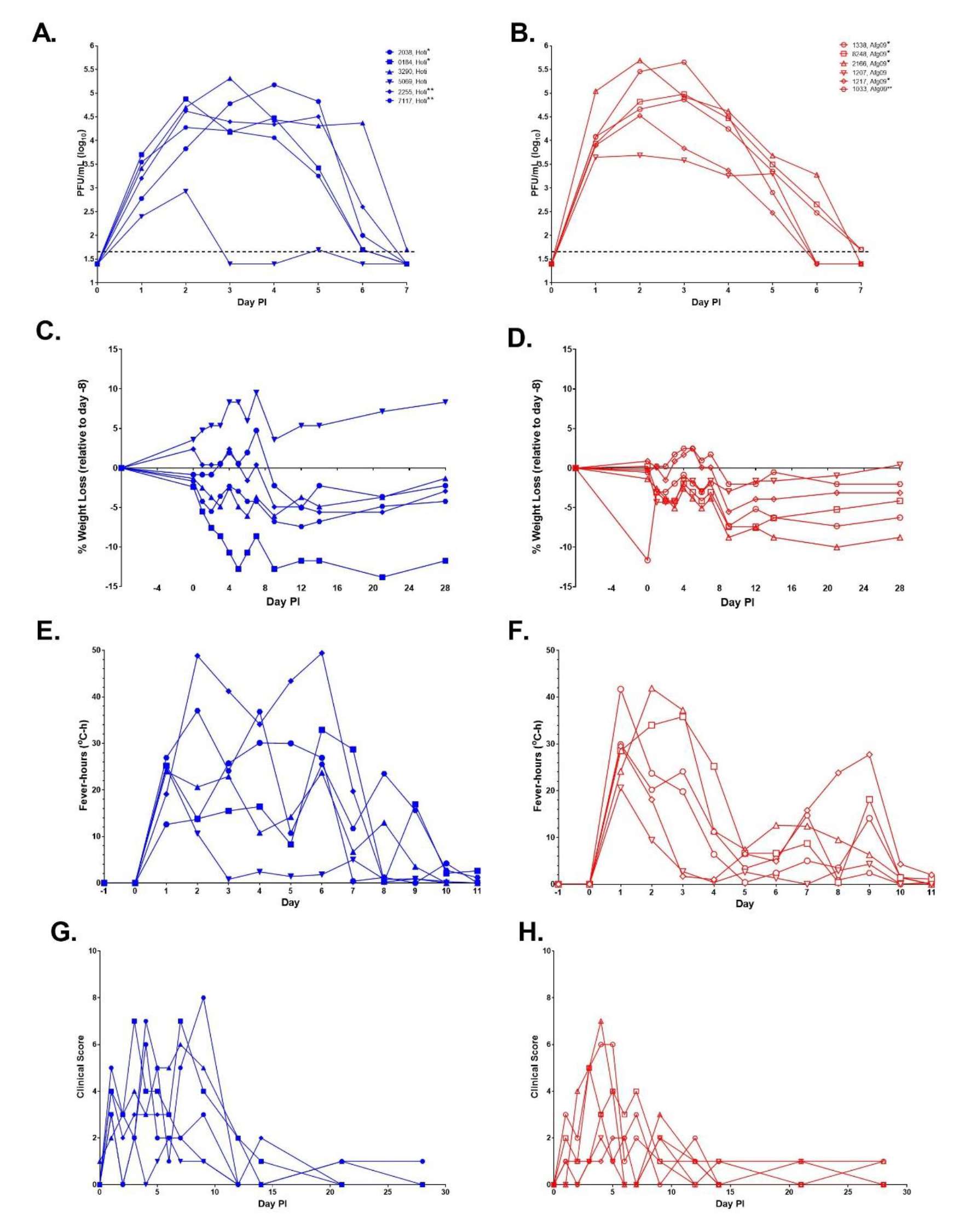

Supplement: S2 Fig — Animals with granulomas detected in the lung, liver, or lymph node are indicated with an asterisk. Animals with orchitis are indicated with two asterisks. A-B) Viremia was determined by standard plaque assay. The dashed line represents the assay LOD. C-D) Weight loss for individual animals shown as percent change in baseline prior to infection. Overall animals infected with both CCHFV strains lost a significant amount of weight compared to baseline values prior to virus exposure (ANOVA; p<0.0001) and there were significant changes over time for both groups (ANOVA; p = 0.0304). E-F) The significant temperature responses are indicated as fever-hours and shown for the individual animals. G-H) Clinical scores were determined on each animal when anesthetized for blood collection. (TIF) [file ppat.1008050.s002.tif]

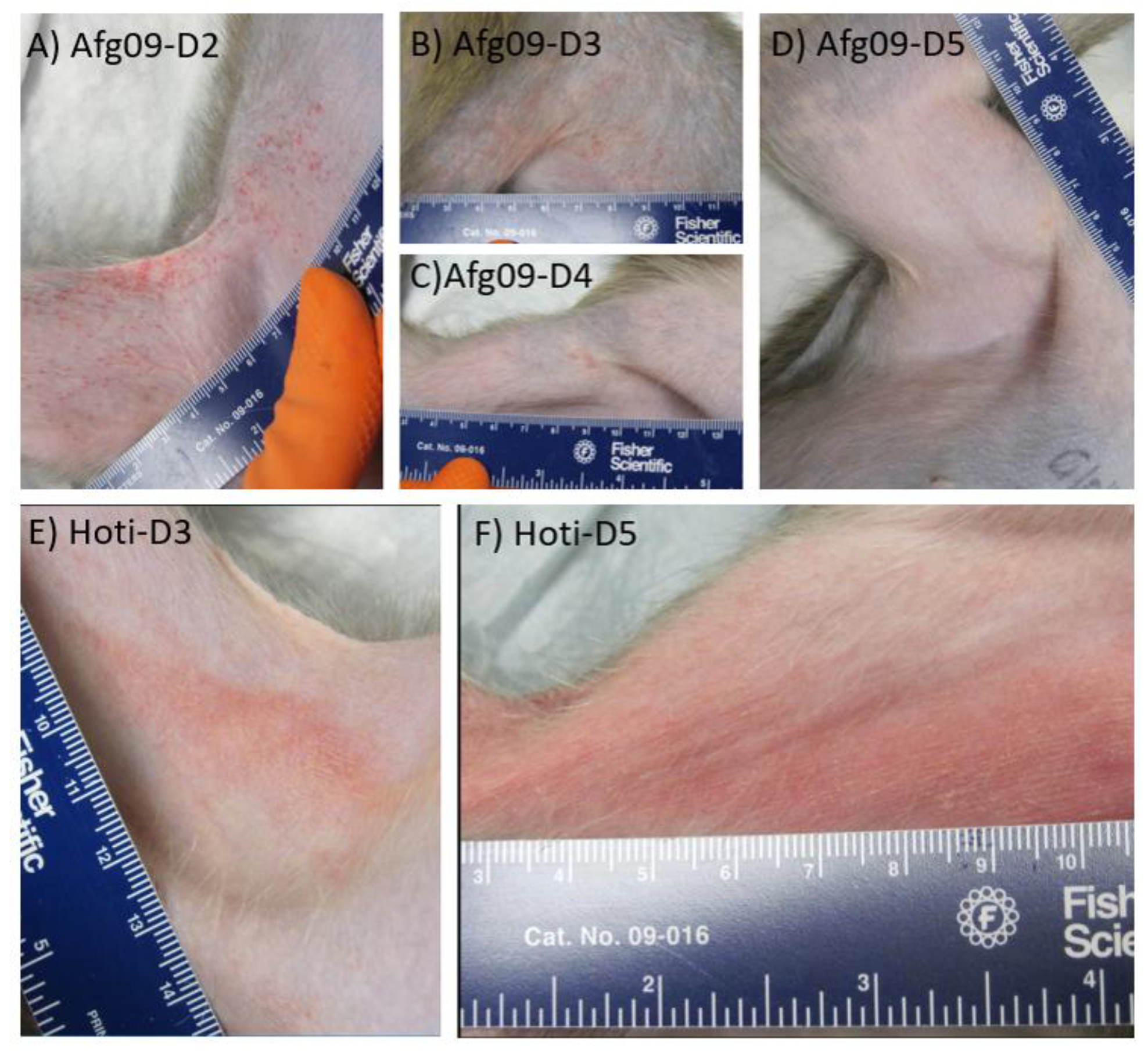

Supplement: S3 Fig — A-D) Development and resolution of petechial rash over a 3 day time period in an NHP infected with Afg09 CCHFV. E-F) Representative images of the macule rash in an NHP infected with Hoti strain CCHFV. Date of image collection is indicated. All photos depict the Axillary region. (TIF) [file ppat.1008050.s003.tif]

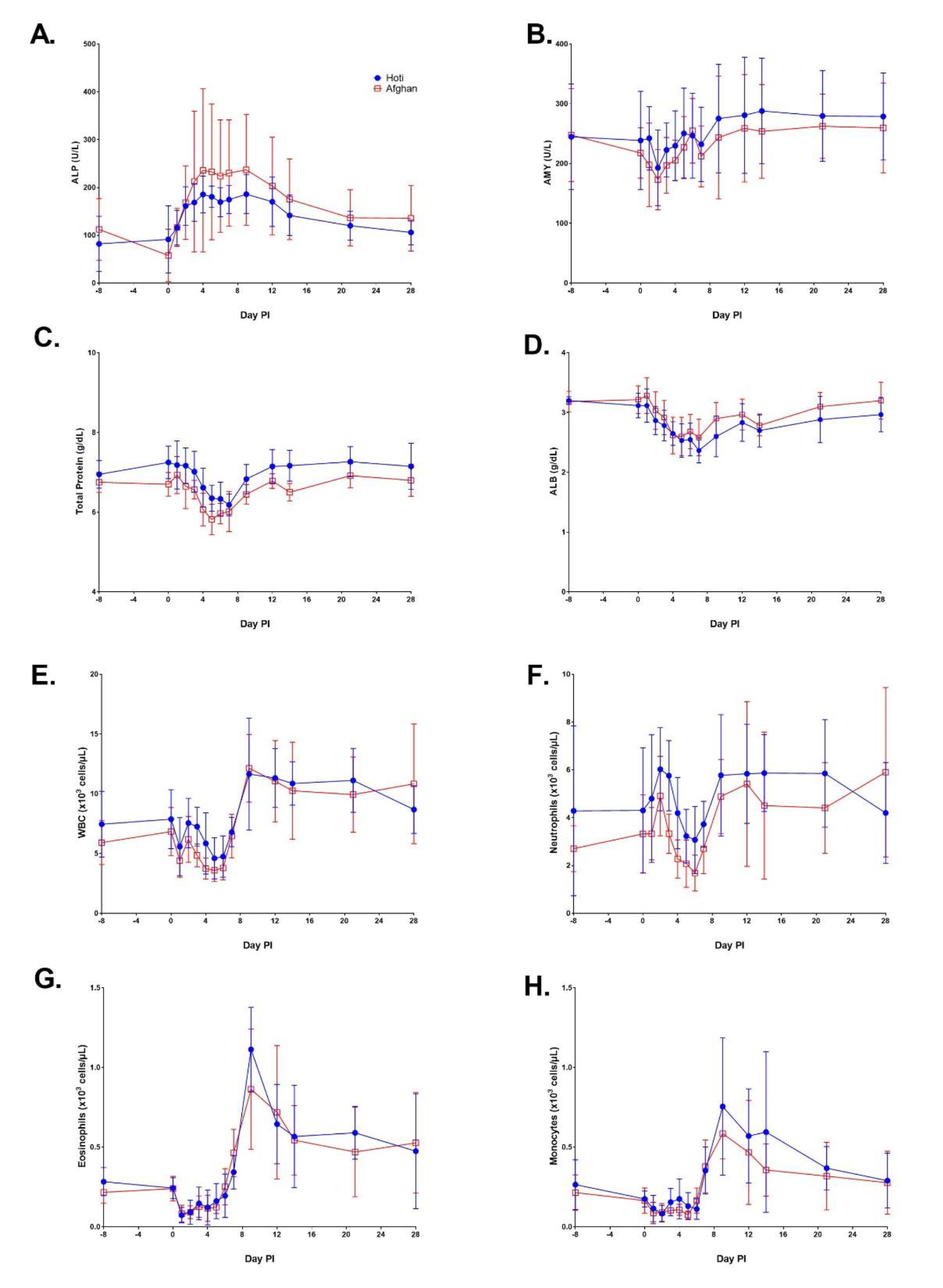

Supplement: S4 Fig — A) ALP. B) AMY. C) Total Protein. D) ALB. E) WBC. F). Neutrophils. G). Eosinophils. H). Monocytes. The symbols represent the mean value and the error bars represent the standard error of the mean. (TIF) [file ppat.1008050.s004.tif]

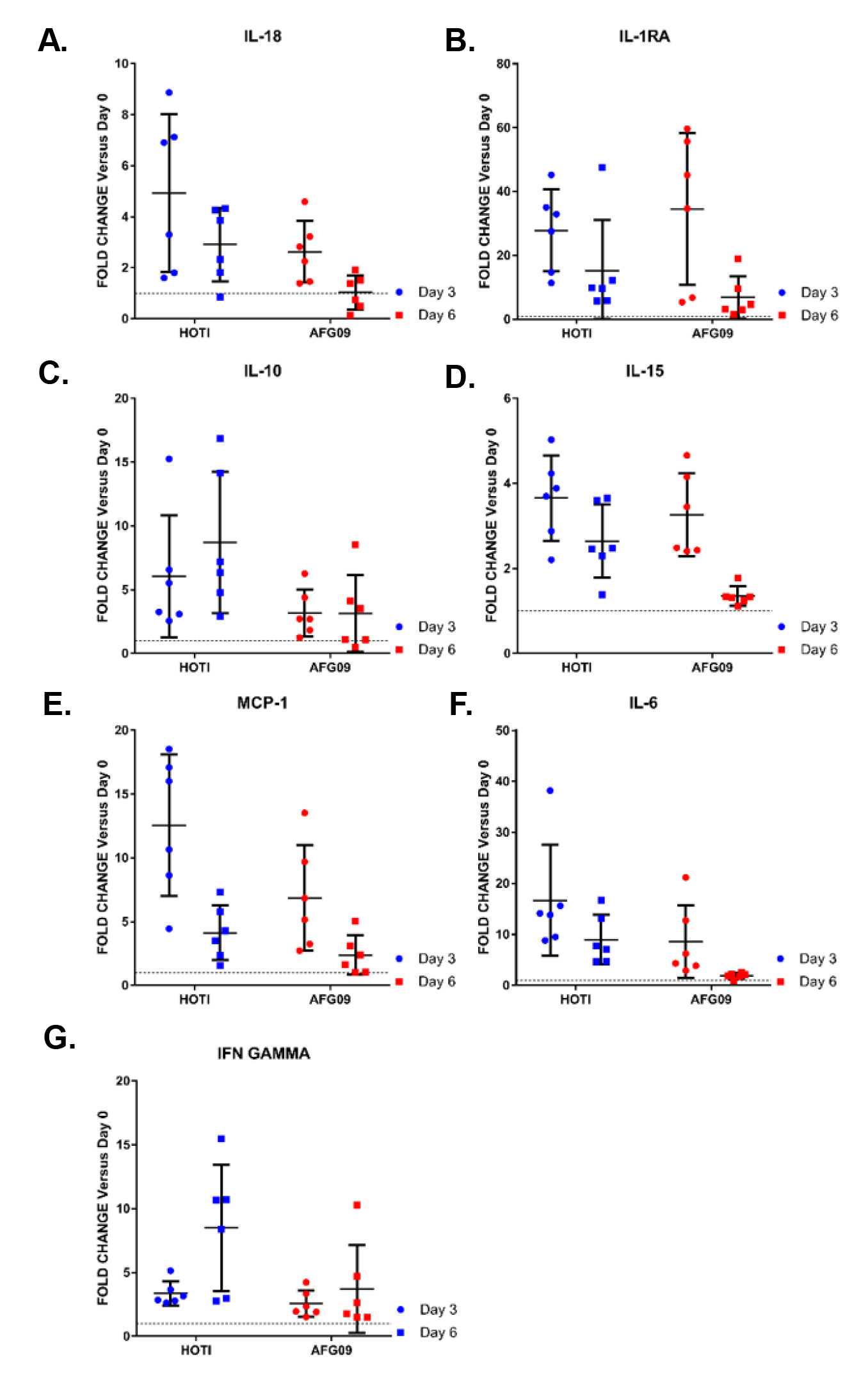

Supplement: S5 Fig — A-G) Day 3 and Day 6 cytokine responses to CCHFV challenge with highest responding cytokines depicted. Serum samples were run in duplicate for each time point. Individual NHP cytokine responses are shown for each time point. Signal values were derived from MFI and depict fold change relative to day 0 for each NHP tested. Geometric means and standard deviations for each group time point are shown along with standard deviation bars. (TIF) [file ppat.1008050.s005.tif]

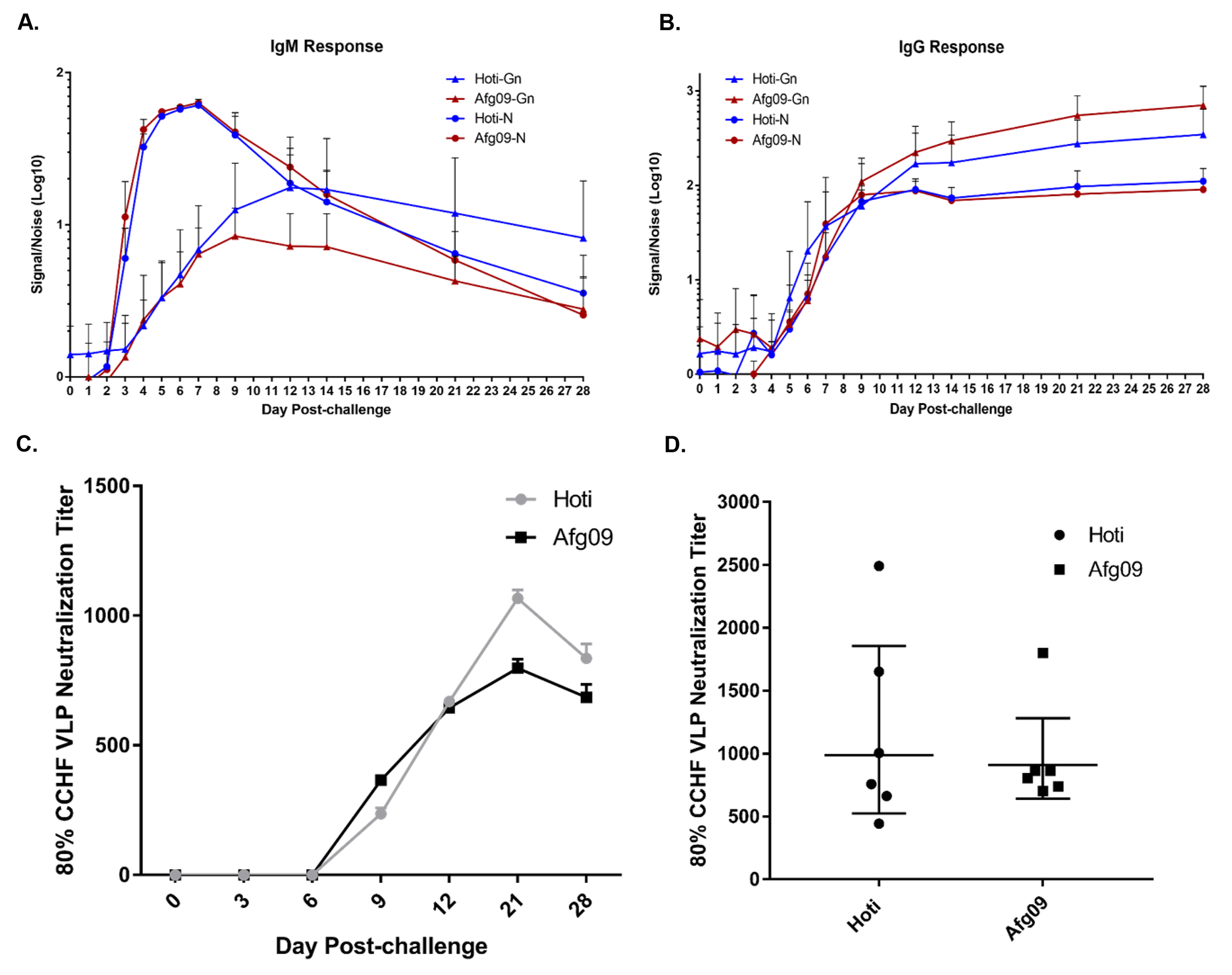

Supplement: S6 Fig — A) IgM and B) IgG responses to N protein and GN protein during acute and convalescent phases of CCHFV infection. All data in A and B represents individual samples run in duplicate and measured for MFI. Signal to noise values were then averaged across the entire group (Hoti or Afg09). C) Time course of neutralizing response of pooled group seras against IbAr 10200 strain CCHF VLPs. D) Individual terminal (Day 28) neutralizing responses of all NHPs challenged in this study, as assessed by inhibition of VLP infection. Pooled naïve NHP sera was used as a positive control while sera from a convalescent human sera was used as a positive control. All neutralizing titers were derived from dilution series run in duplicate. Error bars represent standard deviation for all data sets. (TIF) [file ppat.1008050.s006.tif]
