# Supplementary material for: Persistent Crimean-Congo hemorrhagic fever virus infection in the testes and within granulomas of non-human primates with latent tuberculosis
Source: PLoS Pathog. 2019 Sep 26;15(9):e1008050. doi: 10.1371/journal.ppat.1008050 (PMC6782109; doi:10.1371/journal.ppat.1008050)
Supplement: S1 Table — (DOCX) [file ppat.1008050.s007.docx]

| **Parameter** | **Day Effect** | **Strain Effect** | **Day and Strain Effect** |
| --- | --- | --- | --- |
| ALB | **0.0064** | 0.4516 | 0.4924 |
| ALP | **0.0088** | 0.0846 | 0.8899 |
| ALT | **<0.0001** | 0.4370 | 0.6341 |
| AMY | **<0.0001** | 0.1890 | 0.8406 |
| AST | **<0.0001** | 0.8267 | 0.9627 |
| BUN | 0.4951 | 0.0696 | 0.4306 |
| CA | **<0.0001** | 0.4467 | 0.6627 |
| CRE | **0.0001** | 0.3750 | 0.1600 |
| GGT | **0.0220** | **0.0242** | 0.6898 |
| GLU | **<0.0001** | **0.0098** | 0.1462 |
| TBIL | **0.0007** | 0.5419 | 0.8896 |
| TP | **0.0003** | 0.2768 | 0.8936 |
