# Supplementary material for: Persistent Crimean-Congo hemorrhagic fever virus infection in the testes and within granulomas of non-human primates with latent tuberculosis
Source: PLoS Pathog. 2019 Sep 26;15(9):e1008050. doi: 10.1371/journal.ppat.1008050 (PMC6782109; doi:10.1371/journal.ppat.1008050)
Supplement: S2 Table — (DOCX) [file ppat.1008050.s008.docx]

| **Parameter** | **Day Effect** | **Strain Effect** | **Day and Strain Effect** |
| --- | --- | --- | --- |
| BASO | 0.1202 | 0.0470 | 0.6692 |
| EOS | **<0.0001** | 0.6591 | 0.9160 |
| HB | **<0.0001** | 0.6149 | 0.2217 |
| HCT | **0.0165** | 0.2389 | 0.7880 |
| LYMPH | **<0.0001** | 0.5296 | 0.5387 |
| MCH | **0.0002** | 0.0534 | 0.0631 |
| MCHC | **0.0009** | 0.3841 | 0.1769 |
| MCV | 0.1119 | 0.1594 | 0.0576 |
| MONO | **<0.0001** | 0.8052 | 0.7359 |
| MPV | 0.2721 | 0.7066 | 0.6557 |
| NEUT | **0.0035** | **0.0221** | **0.0297** |
| %BASO | **0.0345** | 0.0848 | 0.7737 |
| %EOS | **<0.0001** | 0.2588 | 0.3695 |
| %LYMPH | **<0.0001** | **0.0026** | 0.0879 |
| %MONO | **0.0388** | 0.4947 | 0.2296 |
| %NEUT | **<0.0001** | **0.0053** | 0.0809 |
| PLT | **<0.0001** | 0.3470 | 0.7741 |
| RBC | **0.0208** | 0.4134 | 0.9706 |
| RDW | **<0.0001** | 0.1256 | 0.3157 |
| WBC | **<0.0001** | 0.2296 | 0.1052 |
